# Supplementary material for: Increasing control over biomineralization in conodont evolution
Source: Nat Commun. 2024 Jun 20;15:5273. doi: 10.1038/s41467-024-49526-0 (PMC11190287; doi:10.1038/s41467-024-49526-0)
Supplement: Supplementary file 1 — Supplementary Information [file 41467_2024_49526_MOESM1_ESM.pdf]

Supplementary Information

For *Increasing control over biomineralization in conodont evolution*

Authors

Bryan Shirley[1,2], Isabella Leonhard[3,4], Duncan J. E. Murdock[5], John Repetski[6], Przemysław Świś[4,7], Michel Bestmann[8], Pat Trimby[9,10], Markus Ohl[2], Oliver Plümper[2], Helen E. King[2], Emilia Jarochowska[2]*
Corresponding author: Emilia Jarochowska, e.b.jarochowska@uu.nl

Affiliations

1. Fachgruppe Paläoumwelt, Friedrich-Alexander-Universität Erlangen-Nürnberg, Erlangen, Germany
2. Department of Earth Sciences, Utrecht University, Utrecht, Netherlands
3. Department of Palaeontology, University of Vienna, Vienna, Austria
4. Institute of Evolutionary Biology, Biological and Chemical Research Centre, Faculty of Biology, University of Warsaw, Warsaw, Poland
5. Oxford University Museum of Natural History, Oxford, UK
6. US Geological Survey-Emeritus, MS 926A National Center, Reston, USA
7. Department of Chemical and Geological Sciences, University of Modena and Reggio Emilia, Modena, Italy
8. Department of Geology, University of Vienna, Vienna, Austria
9. Oxford Instruments, High Wycombe, UK
10. Current address: Carl Zeiss Ltd., Cambridge, UK

Supplementary Figures

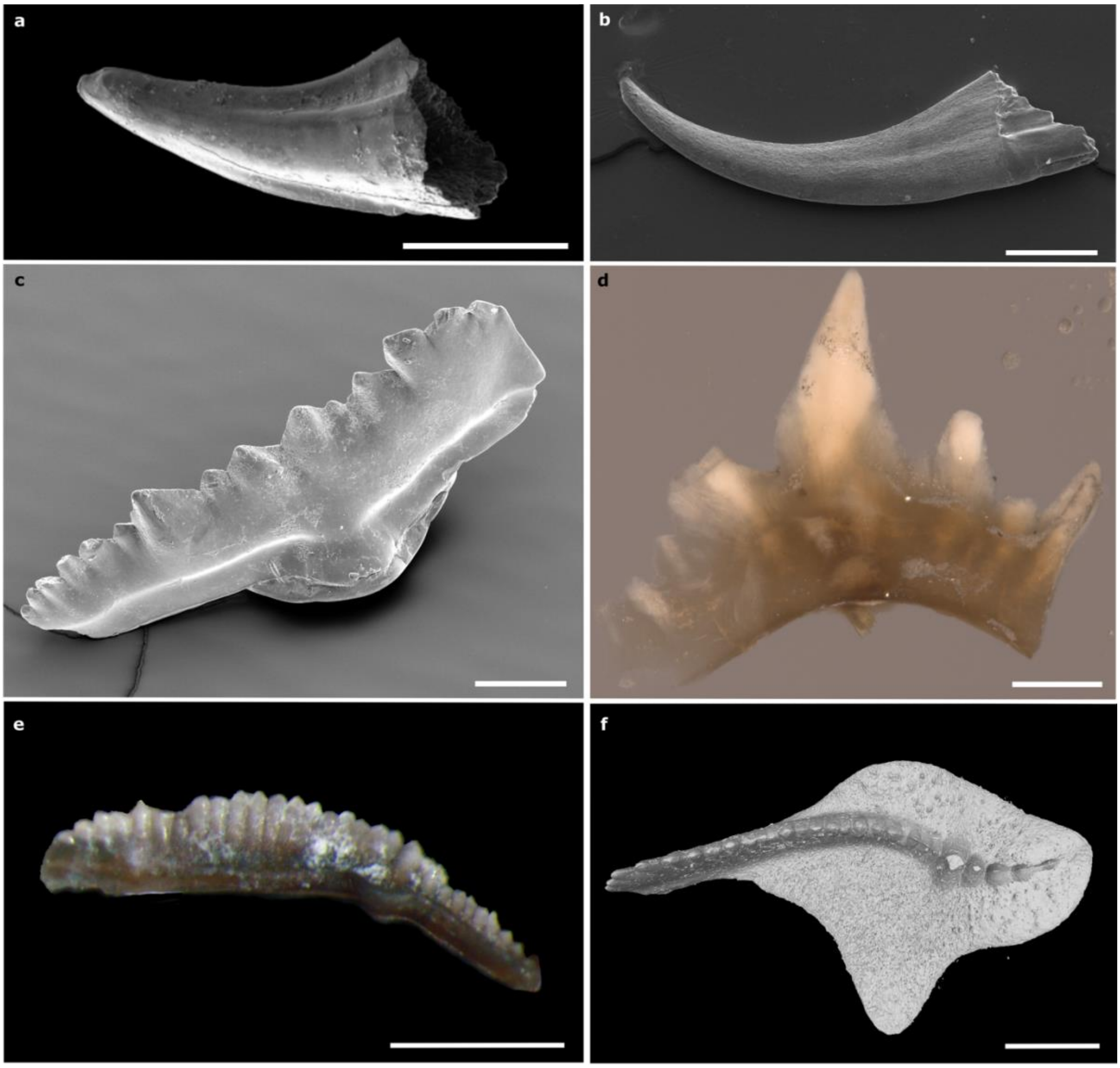

Supplementary Figure 1. Conodont taxa used in the study. a. *Proconodontus muelleri*. b. *Panderodus equicostatus*. c. *Bispathodus* cf. *aculeatus*. d. *Wurmiella excavata*. e. *Tripodellus gracilis*. f. *Palmatolepis* sp. Scale bars 150 µm.

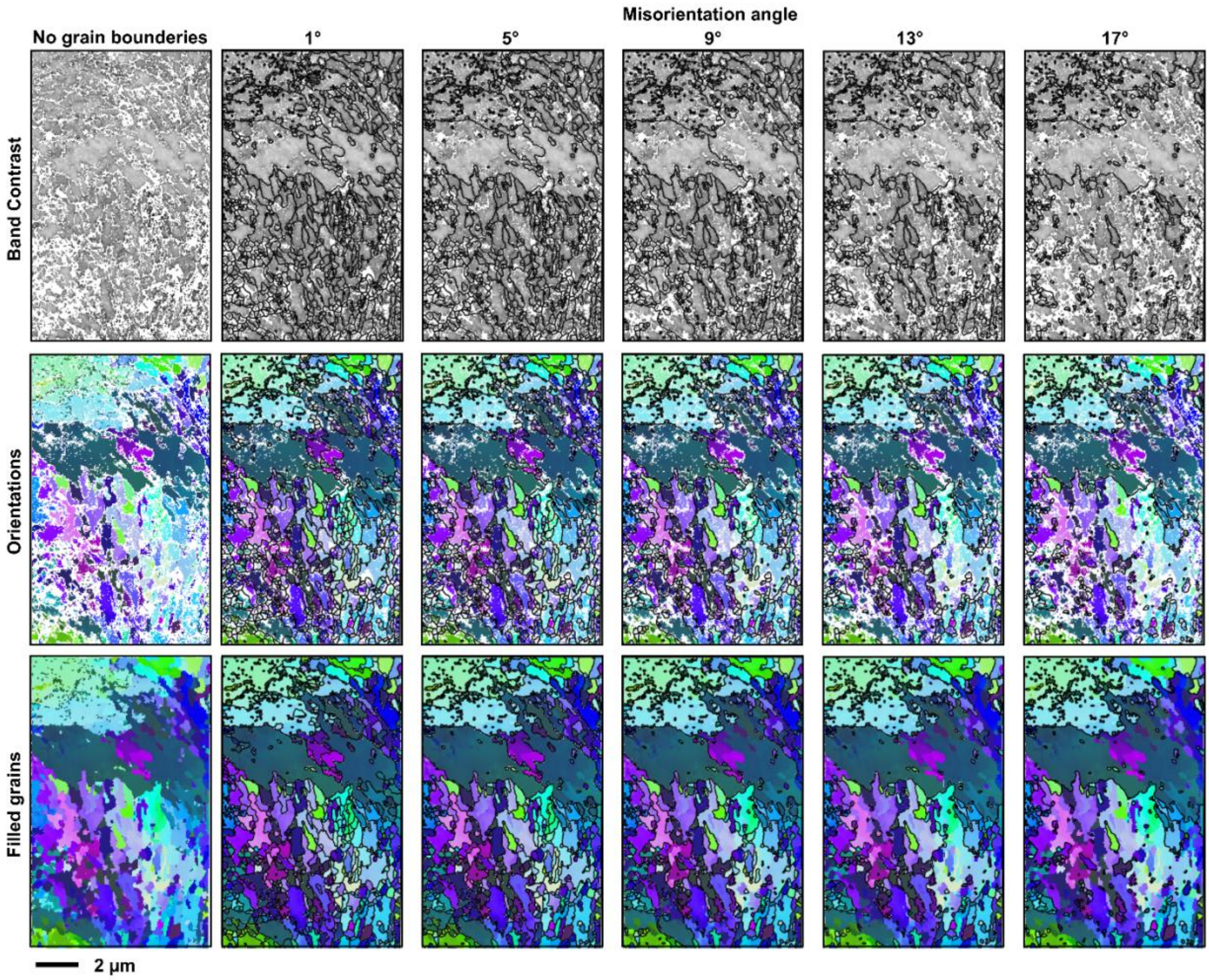

Supplementary Figure 2. Example of the performance of standard grain segmentation algorithms to conodont hyaline tissue, evaluated against band contrast image that reflects the quality of the signal. Grain boundaries are reconstructed using misorientation angles of at 1°, 5°, 9°, 13°, and 17°.

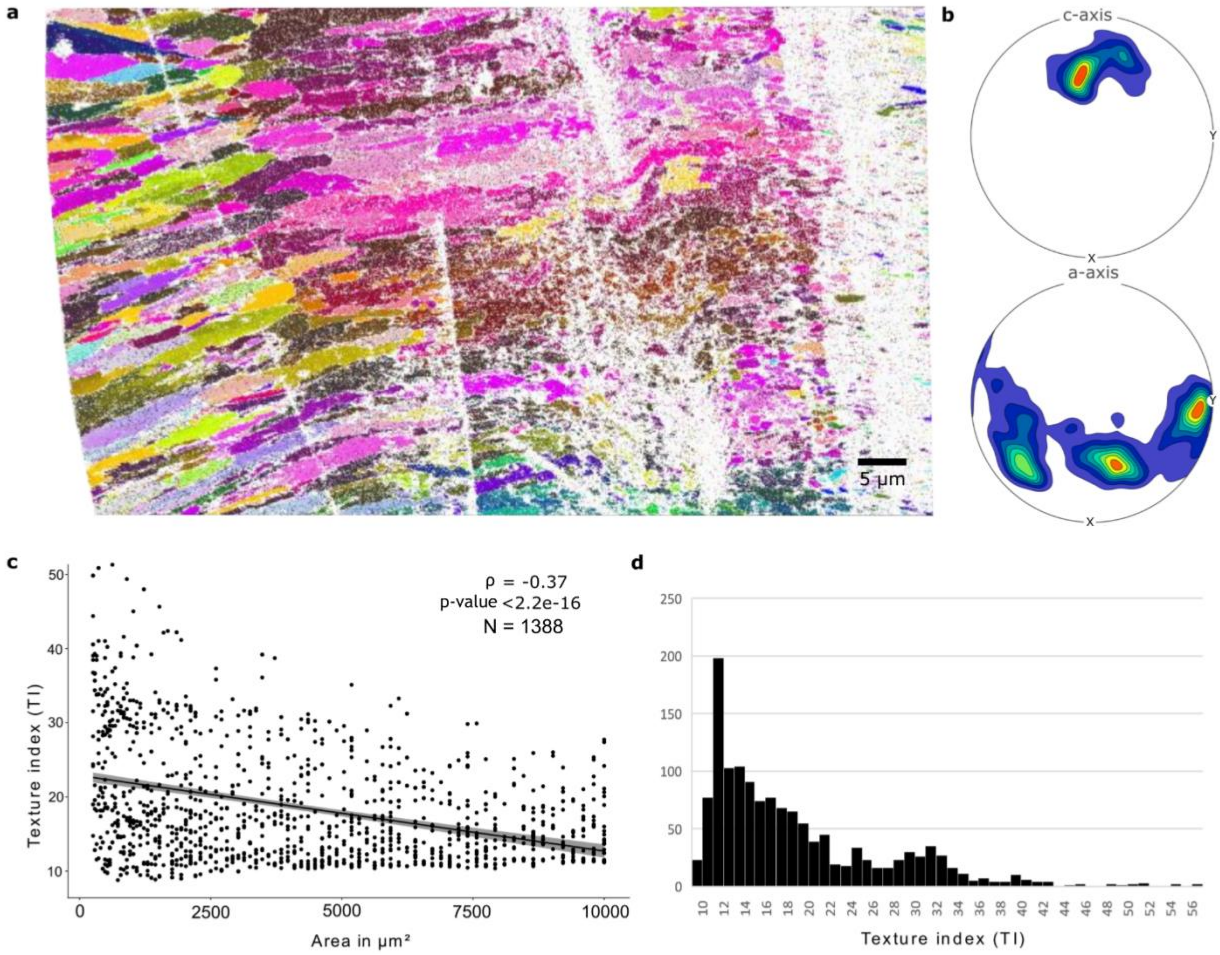

Supplementary Figure 3. The effect of map area on the Texture Index (TI), example illustrated with *Bispathodus* cf. *aculeatus*. **a** EBSD orientation map used for subsampling. **b** Pole figures for the area shown in a. **c** Relationship between the TI and the area obtained using subsampling within the area in a. A least-squares regression line was fitted. N = 1388 subsampling runs. **d** Distribution of TI values across the subsampling iterations. The coordinate system in this sample is as during acquisition[72], without the rotation to align with biological directions, as has been done in **Error! Reference source not found.**. The vertical axis is frequency (counts). N = 1388 subsampling runs.

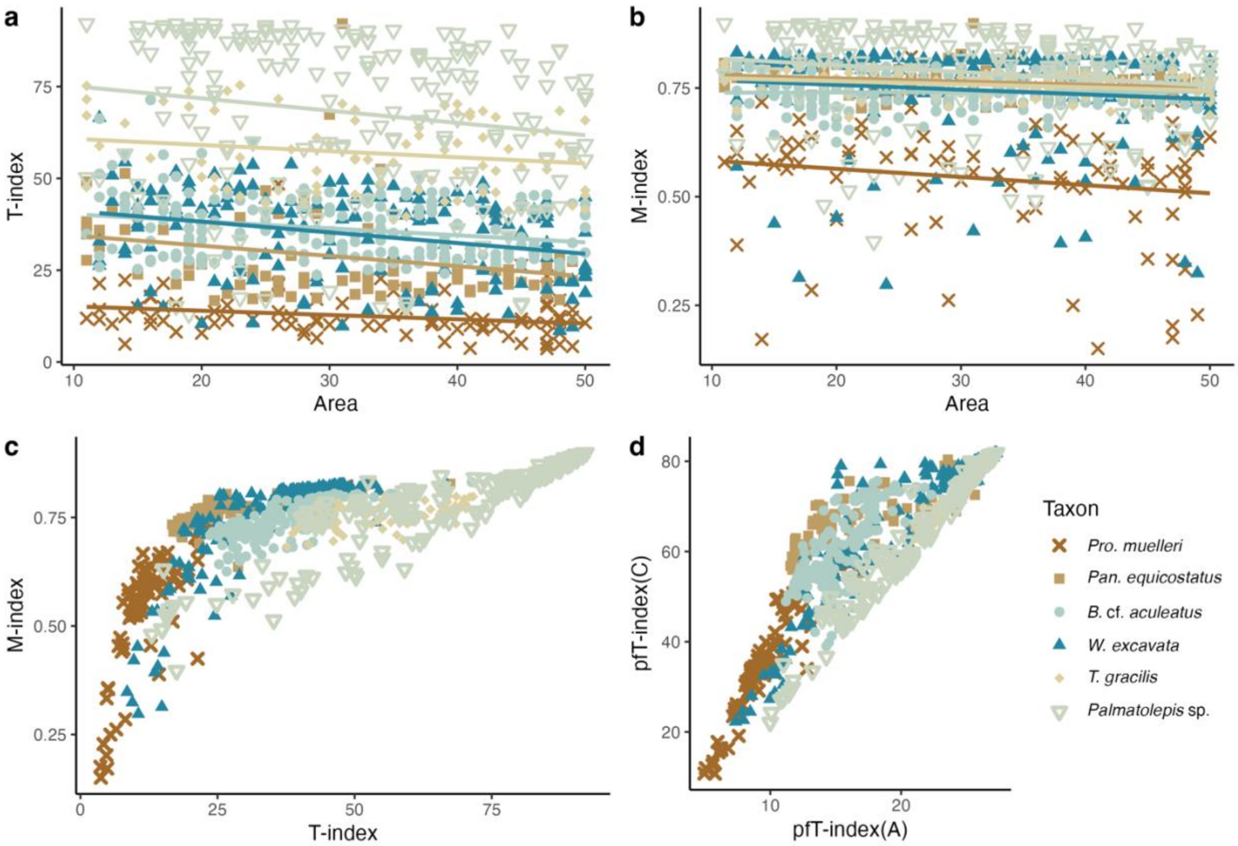

Supplementary Figure 4. Comparison between different texture methods. **a-b** Relationship between index values and the sampled area for Texture Index and the M Index. **c** Direct comparison between the M-index and Texture Index. **d** Relationship of pole figure texture index values between the *a* and *c* axes. N = 797 measurements.

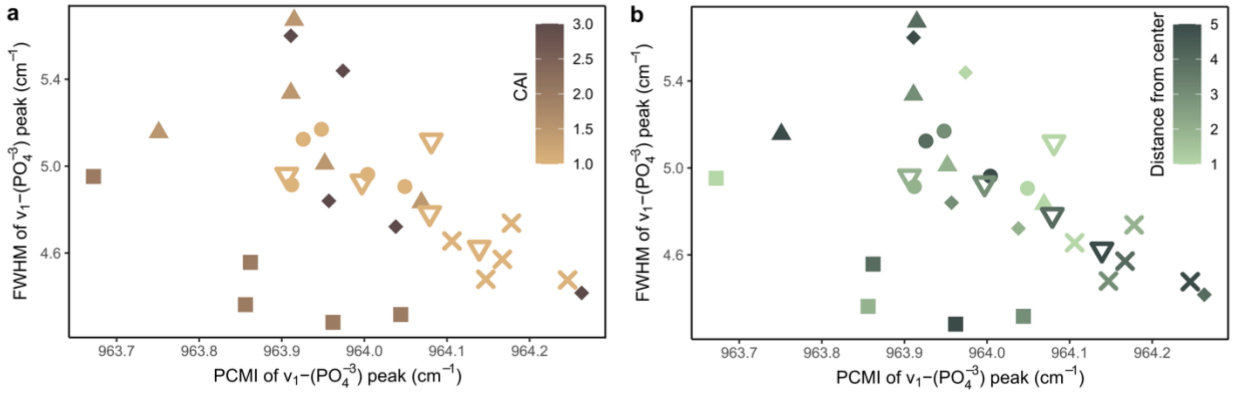

Supplementary Figure 5. Variability of the positions and full width at half maximum (FWHM) of the ν1-PO43− band obtained in Raman analyses across the five measurements for each taxon. a Colored according to the Color Alteration Index (CAI) of the specimen. b Colored according to the distance from the center of each specimen. N = 30 measurements (5 per each of the 6 taxa).

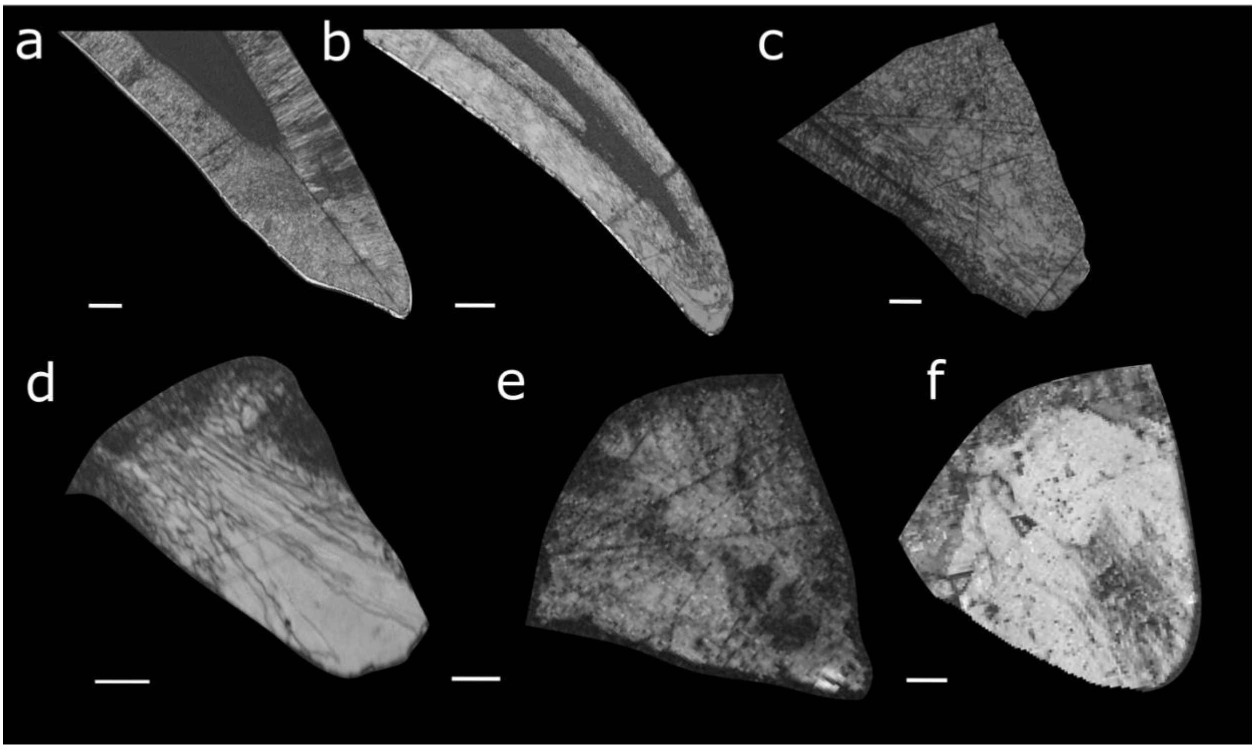

Supplementary Figure 4. Band contrast images representing the quality of diffraction patterns from each EBSD map. **a** *Proconodontus muelleri*, **b** *Panderodus equicostatus,* **c** *Bispathodus* cf. *aculeatus*, **d** *Wurmiella excavata,* **e** *Tripodellus gracilis,* **f** *Palmatolepis* sp. Scale bar 20 µm.

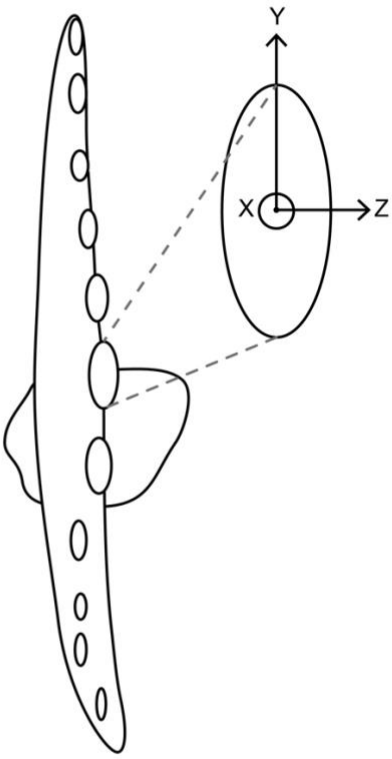

Supplementary Figure 5. Biological directions used to define the coordinate systems in maps shown in Figure 2. The Y direction corresponds to the major axis of the outline of a denticle or cusp. Note that it may correspond to a different position within the apparatus, i.e. with respect to the rest of the animal's body: here the Y direction points towards the ventral side of the animal, but in coniform conodonts the orientation of the element may be different. Thus, the X, Y and Z directions refer to the morphology and the biting function of the tooth, but may differ with respect to how the element was positioned in the body.

Supplementary Tables

Supplementary Table 1. Relationship between analyzed area and Texture Index (TI).

Taxon	Number of subsampled areas	Pearson's correlation coefficient (ρ)	p-value
Wurmiella excavata	246	-0.27	**1.81569×10^{-5}**
Bispathodus* cf. *aculeatus	332	-0.37	**7.5706×10^{-13}**
Panderodus equicostatus	230	-0.42	**1.94788×10^{-11}**
Proconodontus muelleri	184	-0.41	**6.75579×10^{-09}**
**Nautiloid nacre**	342	-0.40	**5.12232×10^{-15}**
**Oyster shell**	465	-0.68	**3.45015×10^{-61}**

Supplementary Table 2. EBSD acquisition parameters.

Taxon	SEM setup	Acceleration voltage [kV]	Probe current [nAmp]	Step size [µm]	Magnification	Number of reflectors (apatite)	Number of reflectors (hydroxylapatite)	Indexing rate [%]
Proconodontus muelleri	Helios NanoLab 600i DualBeam with OI Symmetry	15	22	0.327	1300	72	102	57
Panderodus equicostatus	Helios NanoLab 600i DualBeam with OI Symmetry	15	22	0.532	1599	84	102	63
Bispathodus cf. *aculeatus*	Helios NanoLab 600i DualBeam with OI Symmetry	15	22	0.1	800	48	48	82
Wurmiella excavata	Helios NanoLab 600i DualBeam with OI Symmetry	15	22	0.37	393	48	102	87
Tripodellus gracilis	Zeiss Gemini 350 with OI Symmetry 2	15	15	1	Variable	Variable	Variable	53
Palmatolepis sp.	Zeiss Gemini 350 with OI Symmetry 2	15	15	0.9	Variable	Variable	Variable	54

Supplementary Table 3. Paired Wilcoxon test for the equality of mean values of the Texture Index (TI) between taxa – p-values for the test of the null hypothesis that the location of the means between each pair does not differ from zero. N = 797 measurements.

	Pro. muelleri	***Pan. equicostatus***	***B.* cf. *aculeatus***	***W. excavata***	***T. gracilis***	***Palmatolepis* sp.**
Pro. muelleri		0	0.0000000	0.0000000	0	0
Pan. equicostatus	0		0.0000000	0.0000037	0	0
B.* cf. *aculeatus	0	0		0.6634831	0	0
W. excavata	0	3.7×10^{-6}	0.6634831		0	0
T. gracilis	0	0	0.0000000	0.0000000		2×10^{-7}
***Palmatolepis* sp.**	0	0	0.0000000	0.0000000	2×10^{-7}	

Supplementary Table 4. Paired Wilcoxon test for the equality of mean values of the Texture Index (TI) between taxa – 99.17% confidence interval for the difference in location between TI values for each pair. The confidence intervals are symmetrical at the presented level of precision. N = 797 measurements.

Taxon	*Pro.muelleri*	*Pan.equicostatus*	*B.* cf. *aculeatus*	*W. excavata*	*T. gracilis*	*Palmatolepis* sp.
Pro. muelleri	NA	9,72E-19	7,42E-33	2,18E-24	7,20E-23	6,48E-32
Pan. equicostatus	9,72E-19	NA	2,92E-08	3,67E+00	2,36E-19	5,13E-22
B.* cf. *aculeatus	7,42E-33	2,92E-08	NA	6,63E+05	5,32E-25	2,09E-33
W. excavata	2,18E-24	3,67E+00	6,63E+05	NA	1,03E-21	2,30E-30
T. gracilis	7,20E-23	2,36E-19	5,32E-25	1,03E-21	NA	2,32E-01
***Palmatolepis* sp.**	6,48E-32	5,13E-22	2,09E-33	2,30E-30	2,32E-01	NA
