# Supplementary material for: Increasing control over biomineralization in conodont evolution
Source: Nat Commun. 2024 Jun 20;15:5273. doi: 10.1038/s41467-024-49526-0 (PMC11190287; doi:10.1038/s41467-024-49526-0)
Supplement: Supplementary file 3 — Description of Additional Supplementary Files [file 41467_2024_49526_MOESM3_ESM.pdf]

**Description of Additional Supplementary Files**

File Name: Supplementary  Data 1
Description: Backscatter Electron (BSE) images of examined specimens
