# Supplementary figures and images for: Increasing control over biomineralization in conodont evolution
Source: Nat Commun. 2024 Jun 20;15:5273. doi: 10.1038/s41467-024-49526-0 (PMC11190287; doi:10.1038/s41467-024-49526-0)

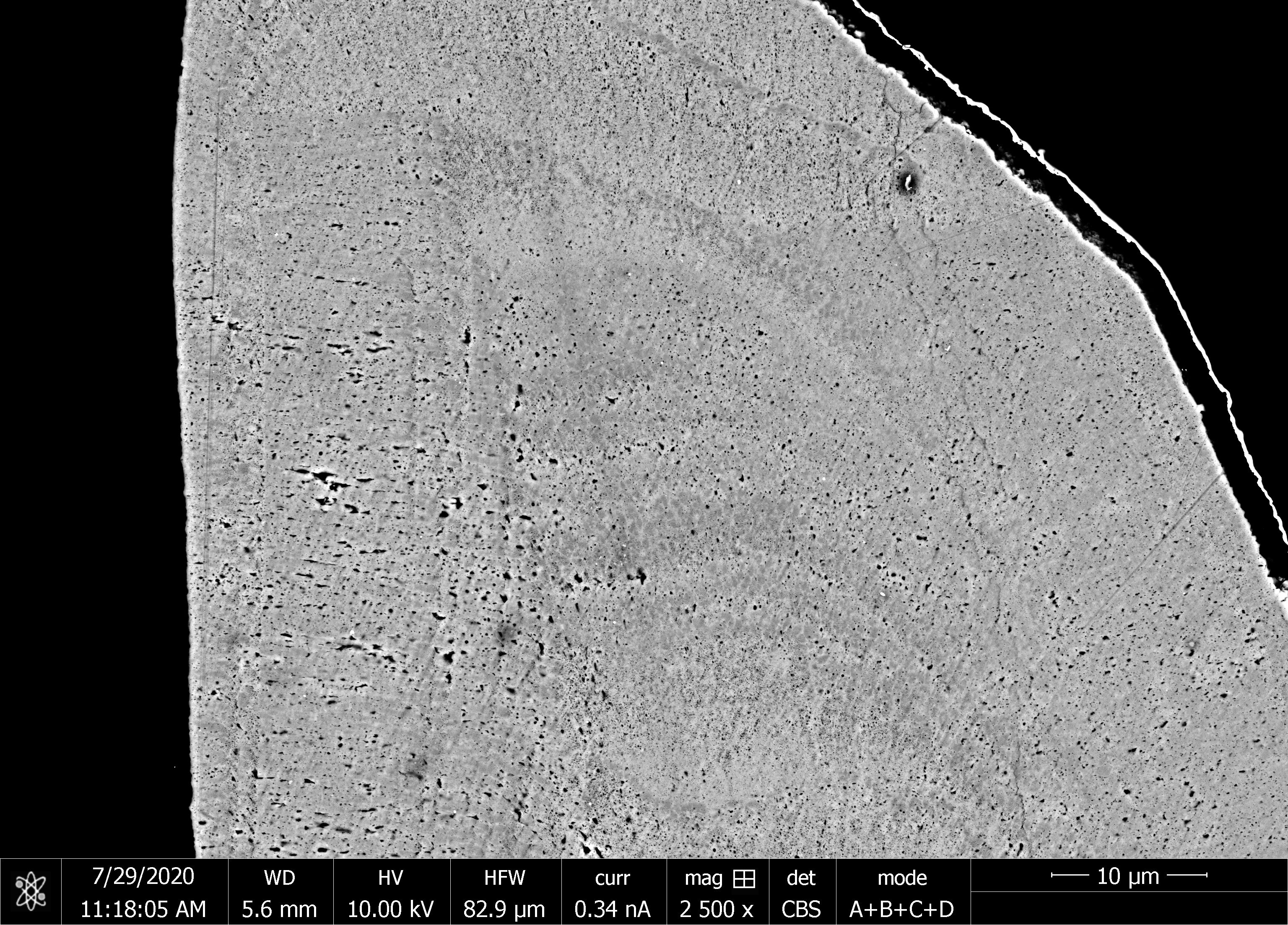

Supplement: Supplementary file 4 — Supplementary Data 1 [file 41467_2024_49526_MOESM4_ESM.zip › Supplementary_Data/Proconodontus_muelleri_2.jpg]

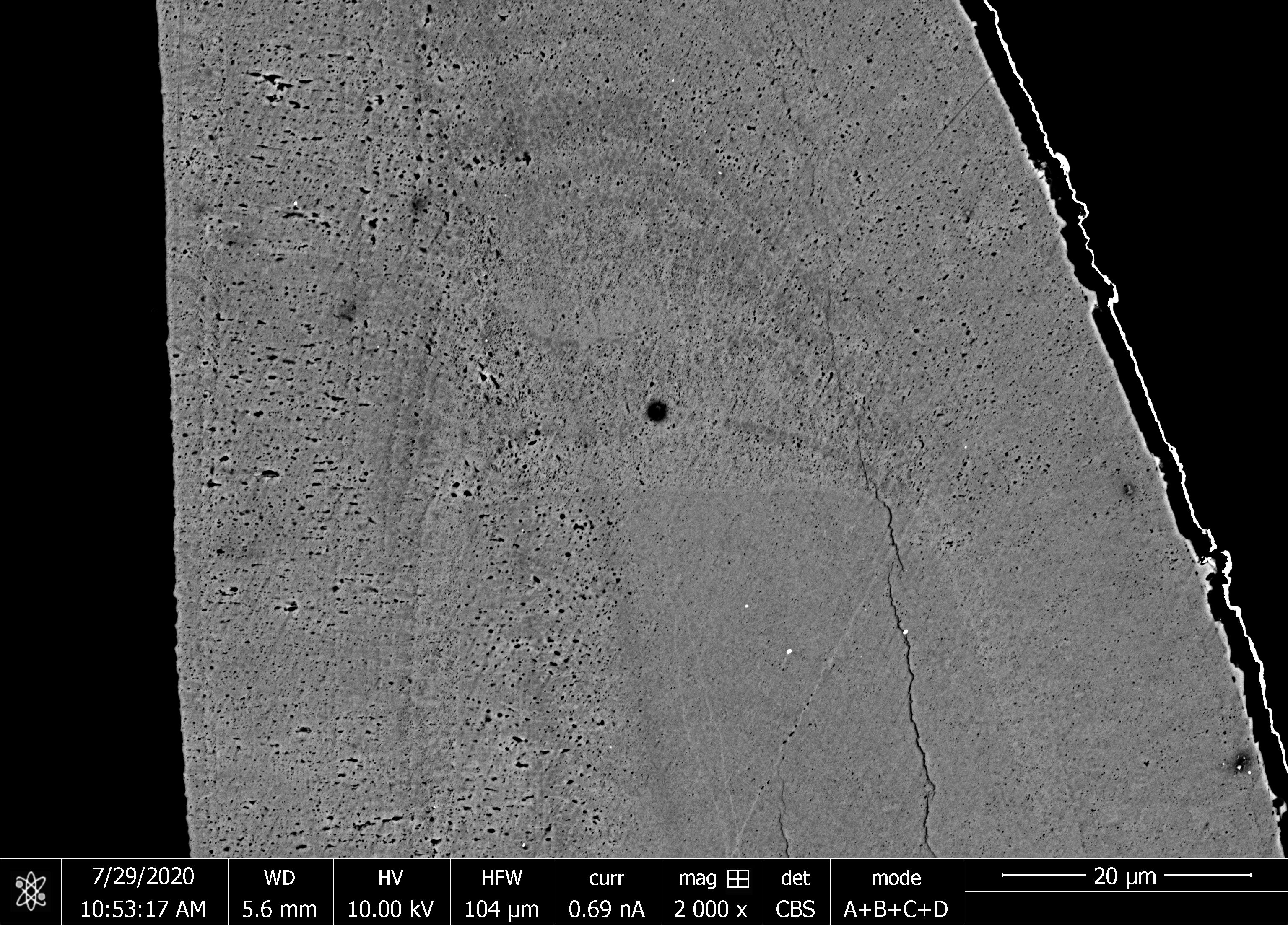

Supplement: Supplementary file 4 — Supplementary Data 1 [file 41467_2024_49526_MOESM4_ESM.zip › Supplementary_Data/Proconodontus_muelleri_1.jpg]

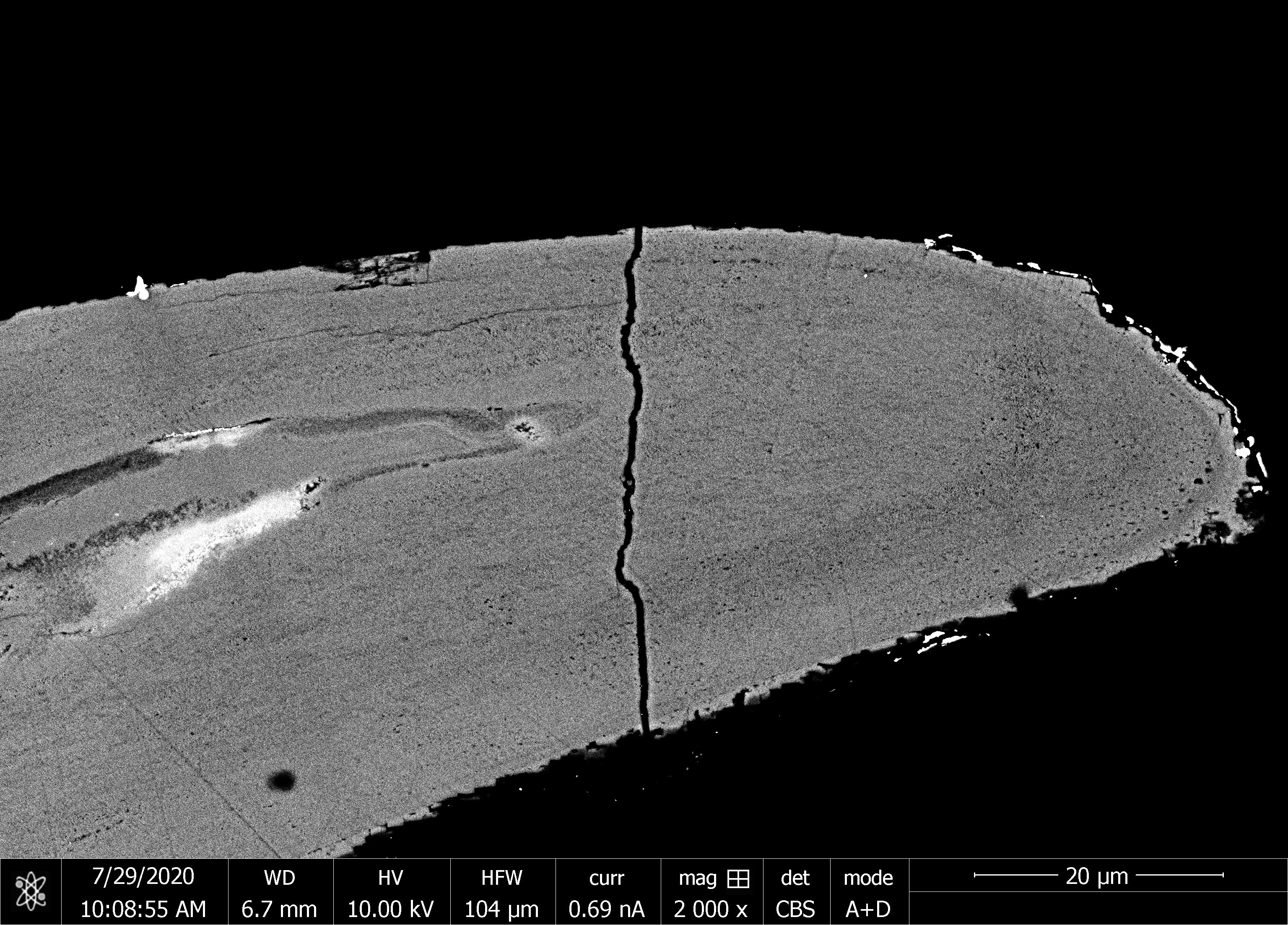

Supplement: Supplementary file 4 — Supplementary Data 1 [file 41467_2024_49526_MOESM4_ESM.zip › Supplementary_Data/Panderodus_equicostatus.jpg]

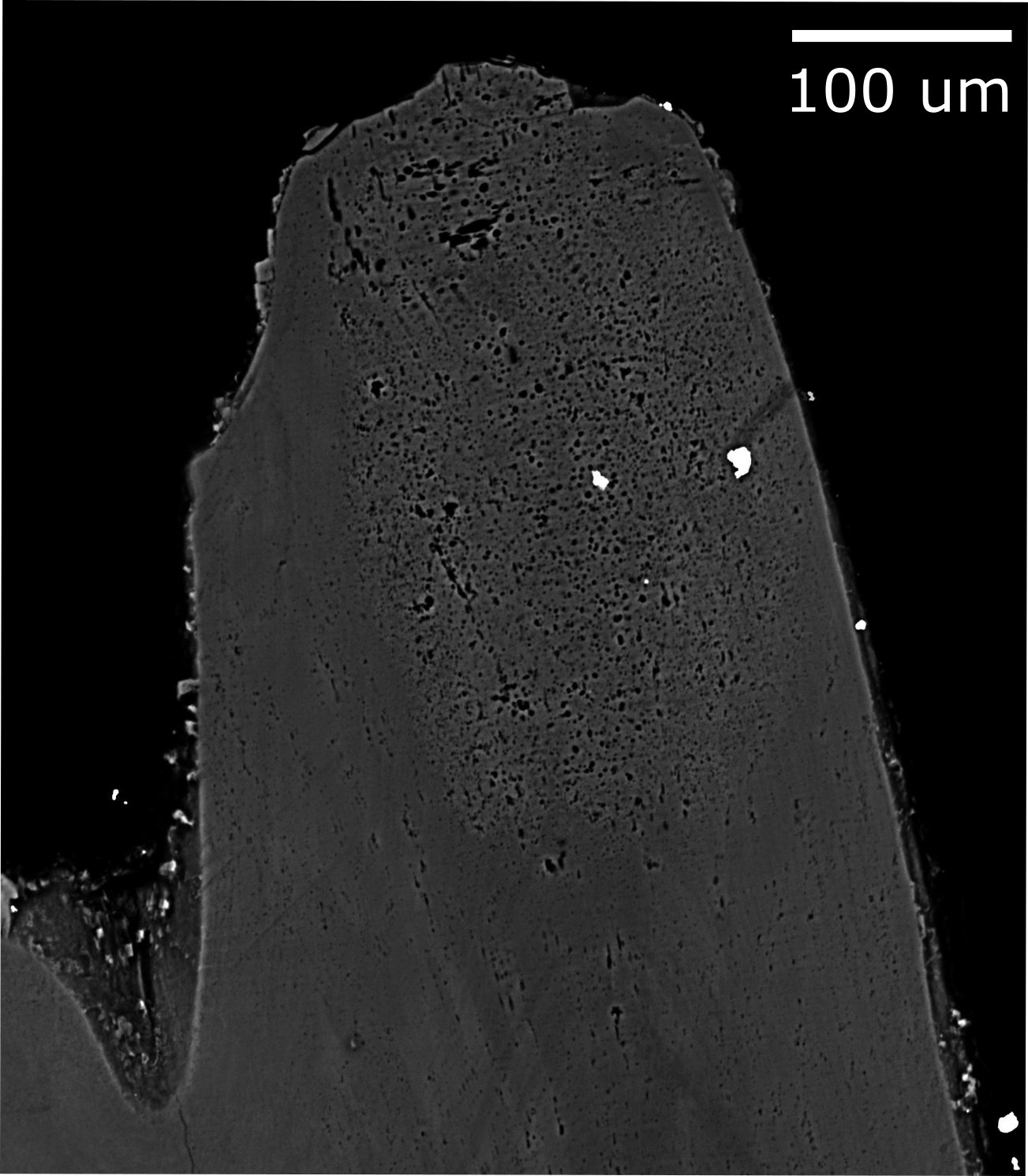

Supplement: Supplementary file 4 — Supplementary Data 1 [file 41467_2024_49526_MOESM4_ESM.zip › Supplementary_Data/Wurmiella_excavata.png]

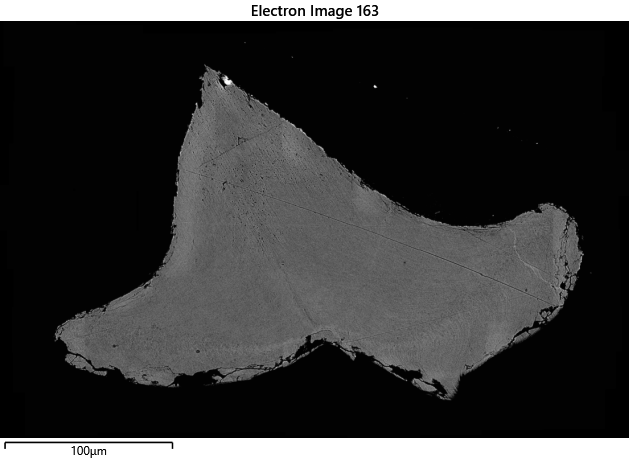

Supplement: Supplementary file 4 — Supplementary Data 1 [file 41467_2024_49526_MOESM4_ESM.zip › Supplementary_Data/Bispathodus_cf._aculeatus.bmp]
