# Supplementary material for: Increasing control over biomineralization in conodont evolution
Source: Nat Commun. 2024 Jun 20;15:5273. doi: 10.1038/s41467-024-49526-0 (PMC11190287; doi:10.1038/s41467-024-49526-0)
Supplement: Supplementary file 5 — Reporting Summary [file 41467_2024_49526_MOESM5_ESM.pdf]

nature portfolio

Corresponding author(s): Emilia Jarochowska

Last updated by author(s): May 20, 2024

Reporting Summary

Nature Portfolio wishes to improve the reproducibility of the work that we publish. This form provides structure for consistency and transparency in reporting. For further information on Nature Portfolio policies, see our Editorial Policies and the Editorial Policy Checklist.

Statistics

For all statistical analyses, confirm that the following items are present in the figure legend, table legend, main text, or Methods section.

n/a	Confirmed	
☐	☒	The exact sample size (n) for each experimental group/condition, given as a discrete number and unit of measurement
☐	☒	A statement on whether measurements were taken from distinct samples or whether the same sample was measured repeatedly
☐	☒	The statistical test(s) used AND whether they are one- or two-sided<br>*Only common tests should be described solely by name; describe more complex techniques in the Methods section.*
☒	☐	A description of all covariates tested
☐	☒	A description of any assumptions or corrections, such as tests of normality and adjustment for multiple comparisons
☐	☒	A full description of the statistical parameters including central tendency (e.g. means) or other basic estimates (e.g. regression coefficient) AND variation (e.g. standard deviation) or associated estimates of uncertainty (e.g. confidence intervals)
☐	☒	For null hypothesis testing, the test statistic (e.g. F, t, r) with confidence intervals, effect sizes, degrees of freedom and P value noted<br>*Give P values as exact values whenever suitable.*
☒	☐	For Bayesian analysis, information on the choice of priors and Markov chain Monte Carlo settings
☒	☐	For hierarchical and complex designs, identification of the appropriate level for tests and full reporting of outcomes
☐	☒	Estimates of effect sizes (e.g. Cohen's d, Pearson's r), indicating how they were calculated

Our web collection on statistics for biologists contains articles on many of the points above.

Software and code

Policy information about availability of computer code

Data collection	commercial: Oxford Instruments AZtec software - EBSD data<br>commercial: Project FIVE 5.2 by WiTec - Raman spectra
Data analysis	Open Source: R Software and own code at https://doi.org/10.5281/zenodo.11222479 (v. 1.0)<br>commercial: MATLAB and own code at https://doi.org/10.5281/zenodo.11222479 (v. 1.0)

For manuscripts utilizing custom algorithms or software that are central to the research but not yet described in published literature, software must be made available to editors and reviewers. We strongly encourage code deposition in a community repository (e.g. GitHub). See the Nature Portfolio guidelines for submitting code & software for further information.

Data

Policy information about availability of data

All manuscripts must include a data availability statement. This statement should provide the following information, where applicable:

- Accession codes, unique identifiers, or web links for publicly available datasets
- A description of any restrictions on data availability
- For clinical datasets or third party data, please ensure that the statement adheres to our policy

Research data is at https://osf.io/c2nu3/ and publicly available

Research involving human participants, their data, or biological material

Policy information about studies with human participants or human data. See also policy information about sex, gender (identity/presentation), and sexual orientation and race, ethnicity and racism.

Reporting on sex and gender	N/A
Reporting on race, ethnicity, or other socially relevant groupings	N/A
Population characteristics	N/A
Recruitment	N/A
Ethics oversight	N/A

Note that full information on the approval of the study protocol must also be provided in the manuscript.

Field-specific reporting

Please select the one below that is the best fit for your research. If you are not sure, read the appropriate sections before making your selection.

☐ Life sciences ☐ Behavioural & social sciences ☒ Ecological, evolutionary & environmental sciences

For a reference copy of the document with all sections, see nature.com/documents/nr-reporting-summary-flat.pdf

Ecological, evolutionary & environmental sciences study design

All studies must disclose on these points even when the disclosure is negative.

Study description	Teeth from 6 fossil taxa (Proconodontus muelleri, Panderodus equicostatus, Palmatolepis sp., Tripodellus gracilis, Wurmiella excavata and Bispathodus cf. aculeatus) were analysed using an SEM microscope to derive quantitative characteristics of their ultrastructure and identify whether these characteristics changed during evolution. Each specimen is meant to represent its taxon. Specimens in already existing collections were used.
Research sample	One tooth from each of the six taxa of the extinct lineage Euconodonta: Proconodontus muelleri, Panderodus equicostatus, Bispathodus cf. aculeatus, Wurmiella excavata, Tripodellus gracilis, Palmatolepis sp. For the last four taxa, homologous tooth positions (P1) were used; for the first two no homology can be established in general. One tooth sample is here assumed to be representative of this homologous position for the respective taxon. The exact age or sex cannot be determined in the case of these fossils.
Sampling strategy	Specimens were selected to represent morphologies corresponding to various feeding modes, to represent the evolution of the ultrastructure towards different material properties. Because of the destructive sample preparation, we excluded specimens of high scientific value (e.g. an important biostratigraphic marker or material illustrated in previous studies) and used common taxa, of which multiple specimens per collection were available. Because of the highly destructive methodology, typically a few specimens were mounted and polished. They were examined under Secondary Electrons, Bascatter Electron, and Band Contrast imaging and those with the largest undamaged areas were used in the study. No prior evaluation of the number of specimens needed was carried out, because data such as collected here had not been collected before and there was no indication of possible variability to allow such calculation.
Data collection	Fossil samples were acquired prior to the study by E. Jarochowska, O. Bremer, J.D. Loch and J.F. Taylor. The initial data (Bispathodus cf. aculeatus) were collected by Patrick Trimby using a Hitachi SU70 FEG SEM. Pro. muelleri, Pan. equicostatus and W. excavata EBSD data was acquired by Bryan Shirley at the Department of Werkstoffwissenschaften WW1 of Friedrich-Alexander-Universität Erlangen-Nürnberg on a Helios NanoLab 600i DualBeam. T. gracilis, Palmatolepis sp. maps were collected by Bryan Shirley and Markus Ohl at the Electron Microscopy Centre of Utrecht University using a Zeiss Gemini 450. All SEMs were fitted with an Oxford Instrument Symmetry EBSD detector using the AZtec software. Raman spectra were collected by Bryan Shirley and Helen E. King on a WiTec alpha 300 Raman microscope at Utrecht University.
Timing and spatial scale	Samples were obtained at various geological localities in Germany, Poland, Sweden, Ukraine and the USA (details listed in the Methods section) and processed using standard methods: dissolving rock in acid, sieving, picking. The processing has been carried out prior to the design of this study as we sourced the specimens from already existing collections. For the study, complete and fully developed (adult) specimens were selected. The geological age of the specimens is the result of choosing taxa representing different feeding ecologies and phylogenetic positions.
Data exclusions	No data was excluded
Reproducibility	Raw data and code have been provided to allow reproducing all steps of the analysis

Randomization	N/A
Blinding	N/A

Did the study involve field work? ☐ Yes ☒ No

Reporting for specific materials, systems and methods

We require information from authors about some types of materials, experimental systems and methods used in many studies. Here, indicate whether each material, system or method listed is relevant to your study. If you are not sure if a list item applies to your research, read the appropriate section before selecting a response.

Materials & experimental systems

n/a	Involved in the study
☒	☐ Antibodies
☒	☐ Eukaryotic cell lines
☐	☒ Palaeontology and archaeology
☒	☐ Animals and other organisms
☒	☐ Clinical data
☒	☐ Dual use research of concern
☒	☐ Plants

Methods

n/a	Involved in the study
☒	☐ ChIP-seq
☒	☐ Flow cytometry
☒	☐ MRI-based neuroimaging

Palaeontology and Archaeology

Specimen provenance	Rock samples were collected on public land and, in the case of Tripodellus gracilis, in a private quarry with permission. Samples containing Proconodontus muelleri were taken by the United States Geological Survey staff at a locality managed by the institution. Sampling of rocks in Germany, Poland, Sweden and Ukraine on public land is allowed without permission, provided that they do not have commercial value. Extraction of fossils was carried out in the laboratories of USGS, University of Warsaw (Poland) and GeoZentrum Nordbayern (Germany).
Specimen deposition	Utrecht University and (for Tripodellus gracilis) Institute of Paleobiology, Polish Academy of Sciences
Dating methods	Dates from previously published literature were used.

☐ Tick this box to confirm that the raw and calibrated dates are available in the paper or in Supplementary Information.

Ethics oversight	No ethical guidance was required - samples were obtained from rock fragments collected in accordance with local regulations. Conodonts, like other microfossils, are very common and the specimens in this study do not represent unique or rare finds. On the contrary, they should be representative for all other specimens of their respective taxon, so the physical specimens used here mostly have value in terms of reproducibility.

Note that full information on the approval of the study protocol must also be provided in the manuscript.

Plants

Seed stocks	N/A
Novel plant genotypes	N/A
Authentication	N/A
